# Source–sink manipulations differentially affect carbon and nitrogen dynamics, fruit metabolites and yield of Sacha Inchi plants

**DOI:** 10.1186/s12870-021-02931-9

**Published:** 2021-03-30

**Authors:** Zhiquan Cai, Tao Xie, Jin Xu

**Affiliations:** 1 Department of Horticulture, Foshan University, Foshan, 528000 China; 2 Key Laboratory of Tropical Plant Resources and Sustainable Use, Xishuangbanna Tropical Botanical Garden, Chinese Academy of Sciences, Mengla, 666303 China; 3 College of Horticulture, Shanxi Agricultural University, Taigu, 030801 Shanxi China

**Keywords:** *Plukenetia volubilis*, Source–sink regulations, Physiological traits, Growth, Reproductive trait, Fruitlet metabolites

## Abstract

**Background:**

Being a promising tropical woody oilseed crop, the evergreen and recurrent plants of Sacha Inchi **(***Plukenetia volubilis* L.) has complex phenology and source–sink interactions. Carbon source–sink manipulations with control and two treatments (reduce source, ca. 10% mature leaf pruning; reduce sink, 10% fruitlet thinning) were conducted on 2.5-year-old field-grown *P. volubilis* plantation during the early-wet season in a seasonal tropical area.

**Results:**

Leaf photosynthetic rate and specific leaf area largely remained unchanged in response to defoliation or defloration. Compared with control, higher N contents on average were observed in both remaining leaves and branches of the defoliated plants, suggesting that N-mobilization was mainly due to the enhanced N uptake from soil. Carbon, but not N, is a source-driven growth process of *P. volubilis* plants, as defoliation reduced the contents of non-structural carbohydrates (especially sugar) in branches, although temporally, whereas defloration increased available C reserve. The seasonal dynamic pattern of fruit ripening was altered by source–sink regulations. Total seed yield throughout the growing season, which depends on fruit set and retention (i.e., number of matured fruit) rather than individual fruit development (size), was slightly increased by defloration but was significantly decreased by defoliation. Compared with control, defloration did not enrich the KEGG pathway, but defoliation downregulated the TCA cycle and carbohydrate and lipid metabolisms in fruitlets after 24 days of the applications of source–sink manipulation.

**Conclusion:**

Carbohydrate reserves serve to buffer sink–source imbalances that may result from temporary adjustment in demand for assimilates (e.g., defloration) or shortfalls in carbon assimilation (e.g., defoliation). Defoliation is disadvantageous for the yield and also for carbohydrate and lipid accumulation in fruits of *P. volubilis* plants. Although more studies are needed, these results provide new insights to the further improvement in seed yield of the strong source-limited *P. volubilis* plants by source/sink manipulations.

**Supplementary Information:**

The online version contains supplementary material available at 10.1186/s12870-021-02931-9.

## Background

Plant growth and reproduction consume large amounts of carbohydrate and nitrogen (N), which are dominantly derived from photosynthate and N absorption from soils, respectively, and/or the remobilization of internal reserves [1, 2]. Although C and N metabolism are strongly interconnected, the storage physiology of both nutrients shows great differences in response to resource-sink regulations [3]. For instance, nonstructural carbohydrates (NSC; mainly sugar and starch), an indicators of a plant’s carbon balance, are stored in all tissues of woody plants, and remobilization of C reserves is controlled by sink strength [4, 5]. In contrast, N storage tends to be concentrated in specific tissues and N remobilization is mainly source driven, depending on the stored N amount [1, 2, 6].

For crops, by coordinating the relationships between source (e.g., mature leaves) and sink (e.g., fruits) and between the vegetative and reproductive growth, leaf/shoot pruning and mechanically or chemically thinning of flowers and/or fruitlets has been successfully applied to improve fruit load and fruit growth (yield) in some temperate and tropical fruit trees [7–9]. However, plant physiological response to source-sink manipulation is complex, depending on species/variety, environmental conditions, and timing and severity of pruning [5, 9, 10]. Fruit set and fruit development are at least related to carbohydrate availability; for a certain woody crop, a suitable fruit set and fruit number per plant owing to properly thinning of flowers and/or fruitlets can avoid an excessive fruit load on plant that may lead to starvation in later stages [11]. But in an exceptional case in the intensively cultivated olive orchards, C reserve pool in woody tissues, acting as an active sink, was not affected by high fruit load; fruit development did not significantly tap the tree’s C reserve pools [12]. On the other hand, the degree to which plant growth and/or reproduction increased will be a function of both the extent of defoliation and the photosynthetic compensatory employed by tree [13–15]. Defoliation has been demonstrated to either no change or a decrease, or even enhance the photosynthetic capacity of the remaining leaves in some woody species (e.g., *Eucalyptus nitens* [16]; *Abies balsamea* [6]). But the transitory compensatory response in leaves generally disappeared when leaf area was rebuilt [16, 17]. At the whole-plant level, responded to leaf or shoot pruning, partition and translocation of photosynthate and N were triggered in perennial plants [6–8]. The increased utilization of C and N reserves stored in the woody tissues supports the productions of new leaves, inflorescence induction, and plant development, which are essential for a sustainable crop production in term of internal source:sink balance [5]. Moreover, the fruits (seeds) metabolites define levels of ripening, playing an important role in early performance of species [2, 18, 19]. As a diagnostic tool, metabolomics provides a powerful means to gain a better understanding of the physiological responses to environmental stimuli (abiotic and biotic stresses) [20]. For instance, different N metabolites in seeds between two contrasting quinoa (*Chenopodium quinoa*) landraces determined a differential nutrition competitiveness, which have developed differential adaptive responses under the low soil-N conditions [19]. On the other hand, the regulation of metabolic dynamics between primary and secondary metabolites affected the whole-plant performance during various developmental stages, including sink strength, seed formation and fruit ripening [3, 21, 22].

Sacha Inchi (*Plukenetia volubilis* L.), a tropical fast-growing evergreen liana, is a promising oilseed crop [23, 24]. Being highly plastic responded to environmental factors, seed yield and quality of *P. volubilis* plants largely depend on the suitable agricultural practices [25–27]. *P. volubilis* plants flower continuously, with peak mature fruits (seeds) occurring in the late-wet season and dry season in southeast China [26, 27]. In the dry season in the seasonal tropical area, female/fruit number and fruit growth of *P. volubilis* plants could be limited by carbohydrate and N availabilities owing to decrease of photosynthates and reduction in cell turgor under drought. In addition, as a liana species, *P. volubilis* plants form a flat canopy with abundant leaves; the over-vegetative growth, especially in the wet season, may result in decline in flowering and fruit (seed) production. But, until now, no study has explored the mechanisms of *P. volubilis* plants under variable source–sink regulations at physiological and metabolomic levels.

An integrated understanding of physiological traits, growth, and reproductive traits (including metabolites) in response to source–sink regulations is a critical step to increase seed production. In the present work, a field experiment was done to elucidate physiological and yield traits in response to defoliation (reduce source) or defloration (reduce sink), which were applied in an intensively cultivated *P. volubilis* plantation in the early-wet season. Specifically, we asked the following questions: (1) Which component of source and sink within plant system and the underlying physiological mechanisms exert the stronger effect over plant growth and/or seed yield? (2) Does defoliation reduce plant growth accompanied by a reduction in C and/or N reserves? and what are the differences between C and N reserves in response to resource-sink regulations? (3) What are metabolomic adjustments in fruitlets in response to source or sink regulation?

## Results

### Leaf traits

**Fig. 1:**
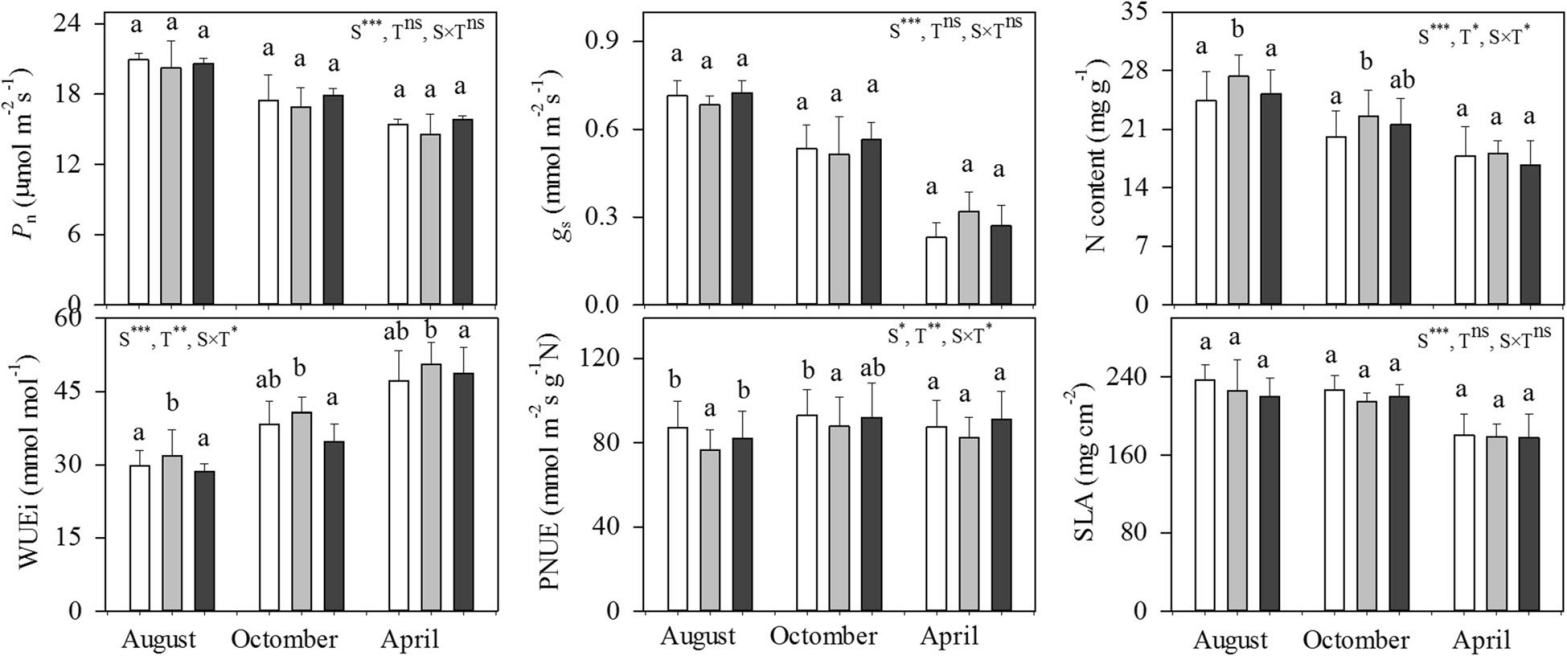
Seasonal variations in leaf morphological and physiological traits variables (means±SD; *n* = 4–6) of *P. volubilis* plants in response to defoliation and defloration. Significance levels of ANOVAs testing for the effects of season (S), treatment (T) and their interaction are listed for each variable. The values with different letters within the same season denote significant difference between different treatments at *P* < 0.05 level. CK, control; ns, no significance. *P < 0.05; **P < 0.01; ***P < 0.001. SLA, specific leaf area; *P*_n_, net light-saturated photosynthetic rate; g_s_, stomatal conductance; WUEi, intrinsic water-use efficiency; PNUE, photosynthetic N-use efficiency

Except for the photosynthetic rate (*P*_n_) and stomatal conductance (g_s_), there were significant season × treatment interactions for the measured leaf physiological traits (Fig. 1a-f), implying that the responsiveness to defoliation or defloration differed between different seasons. For example, defoliation led to a significant increase in leaf N content in the middle-wet season (August), but had no effects in the later seasons (Fig. 1c).

*P*_n_, g_s_, leaf N content and photosynthetic N-use efficiency (PNUE) were highest and lowest in the middle-wet season and the dry season (April), respectively; but the instantaneous water-use efficiency (WUEi) was highest in the dry season across all treatments (Fig. 1). Except for *P*_n_ and gs, defoliation or defloration treatment significantly affected the measured leaf traits. Compared with control, defoliation did not affect *P*_n_ and *g*_s_ in each season, but generally increased N content and WUEi across all seasons (Fig. 1c, d). In contrast, except for a slightly higher leaf N content, defloration had no significant effects on other variables compared with control. With the lowest value in the dry season, SLA did not change significantly between different source–sink regulations (Fig. 1f).

### Carbohydrates and N in branch

**Fig. 2:**
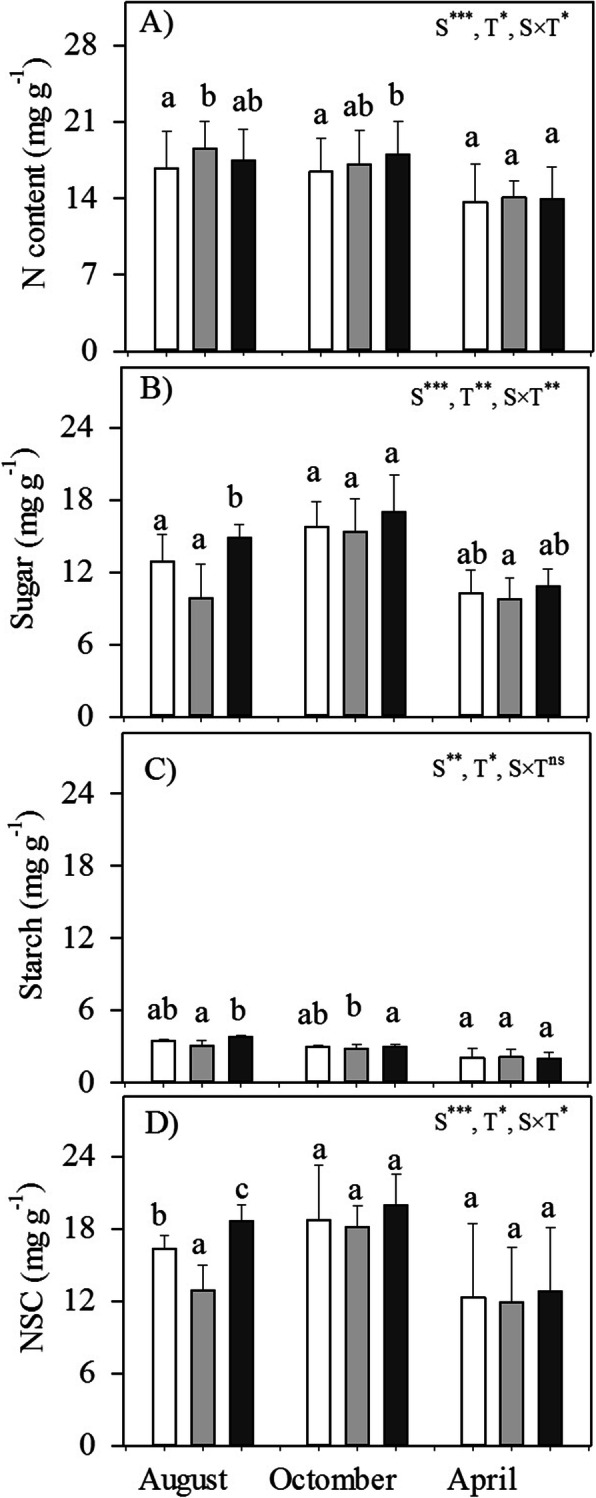
Seasonal variations of the nitrogen (N) content, soluble sugar and starch content, and total nonstructural carbohydrate content (NSC) in branches (means±SD; *n* = 4–5) of *P. volubilis* plants in response to defoliation and defloration. Significance levels of ANOVAs testing for the effects of season (S), treatment (T) and their interaction are listed for each variable. The values with different letters within the same season denote significant difference between different treatments at *P* < 0.05 level. CK, control; ns, no significance. * *P* < 0.05; **P < 0.01; ***P < 0.001

The highest and lowest N and NSC contents in branch occurred in the middle-wet and dry season, respectively (Fig. 2a, d). In response to defoliation, although a loss of nutrition reserves (e.g., N and P) in leaves occurred, N content in branch increased (Fig. 2a). Relative with sugar, starch content had lower plasticity in response to different treatments and seasonal variation (Fig. 2b, c). NSC contents, especially sugar, sharply decreased after defoliation applied ca. three weeks (August), but were fully recovered in the late-wet season (October) and were maintained in the dry season. In contrast, defloration led to a higher NSC content than the control only in the the middle-wet season (Fig. 2d).

### Reproductive traits and plant growth

**Fig. 3:**
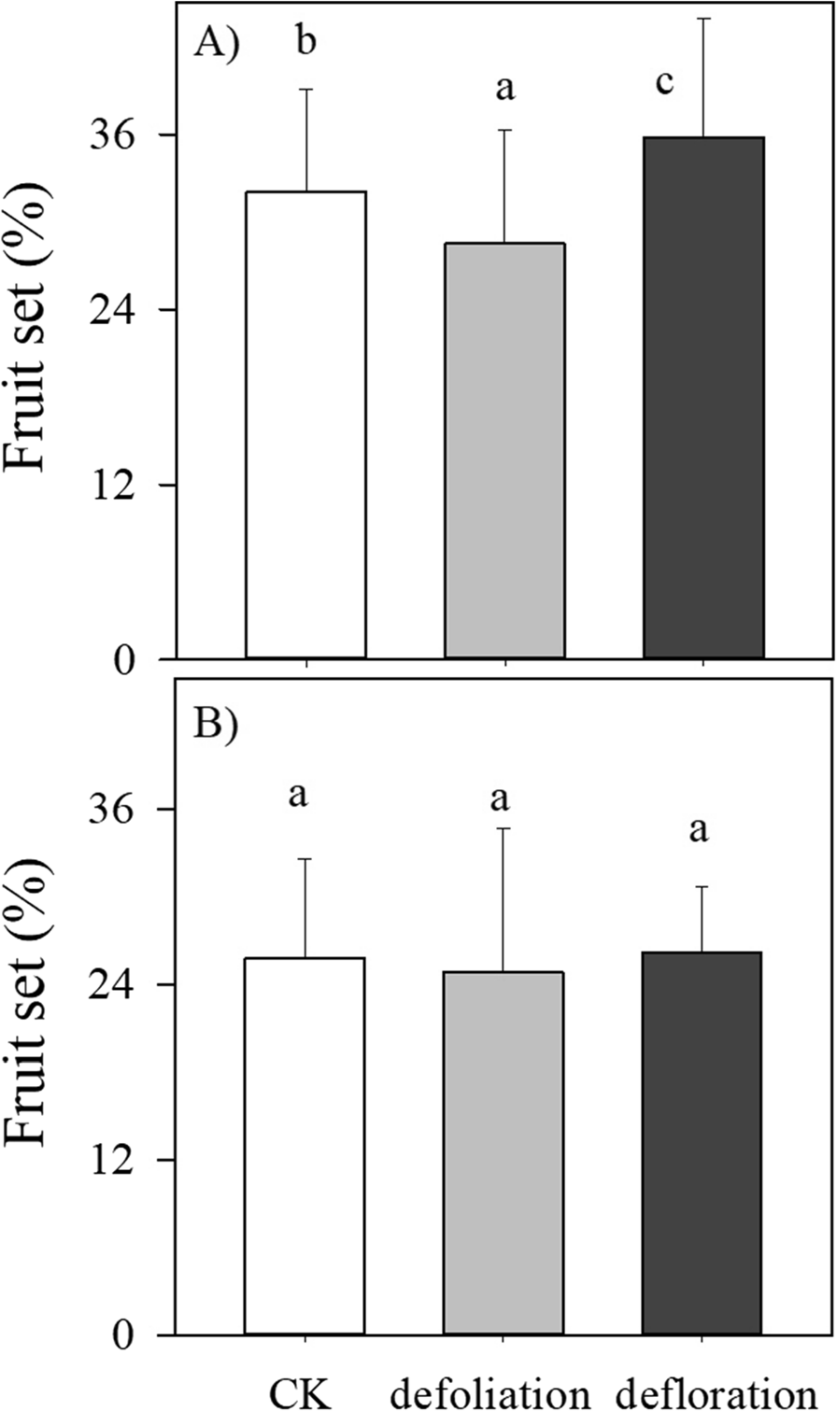
Seasonal variations in the percentage of fruit set (means±SD; *n* = 10) in *P. volubilis* plants in response to defoliation and defloration. **a** female flowers were tagged in late Nov. 2016. **b** female flowers were tagged in Jan. 2017. The values with different letters within the same season denote significant difference at *P* < 0.05 level. CK, control; ns, no significance. * *P* < 0.05; **P < 0.01; ***P < 0.001

Both season and treatment significantly affected the percentage of fruit set (Fig. 3a, b). Fruit set in the latter stage was lower than that in the former stage (*F* = 0.37, *P* < 0.05). Compared with control, defoliation decreased, but defloration increased the percentage of fruit set, especially in the earlier stage (Fig. 3a, b).

**Fig. 4:**
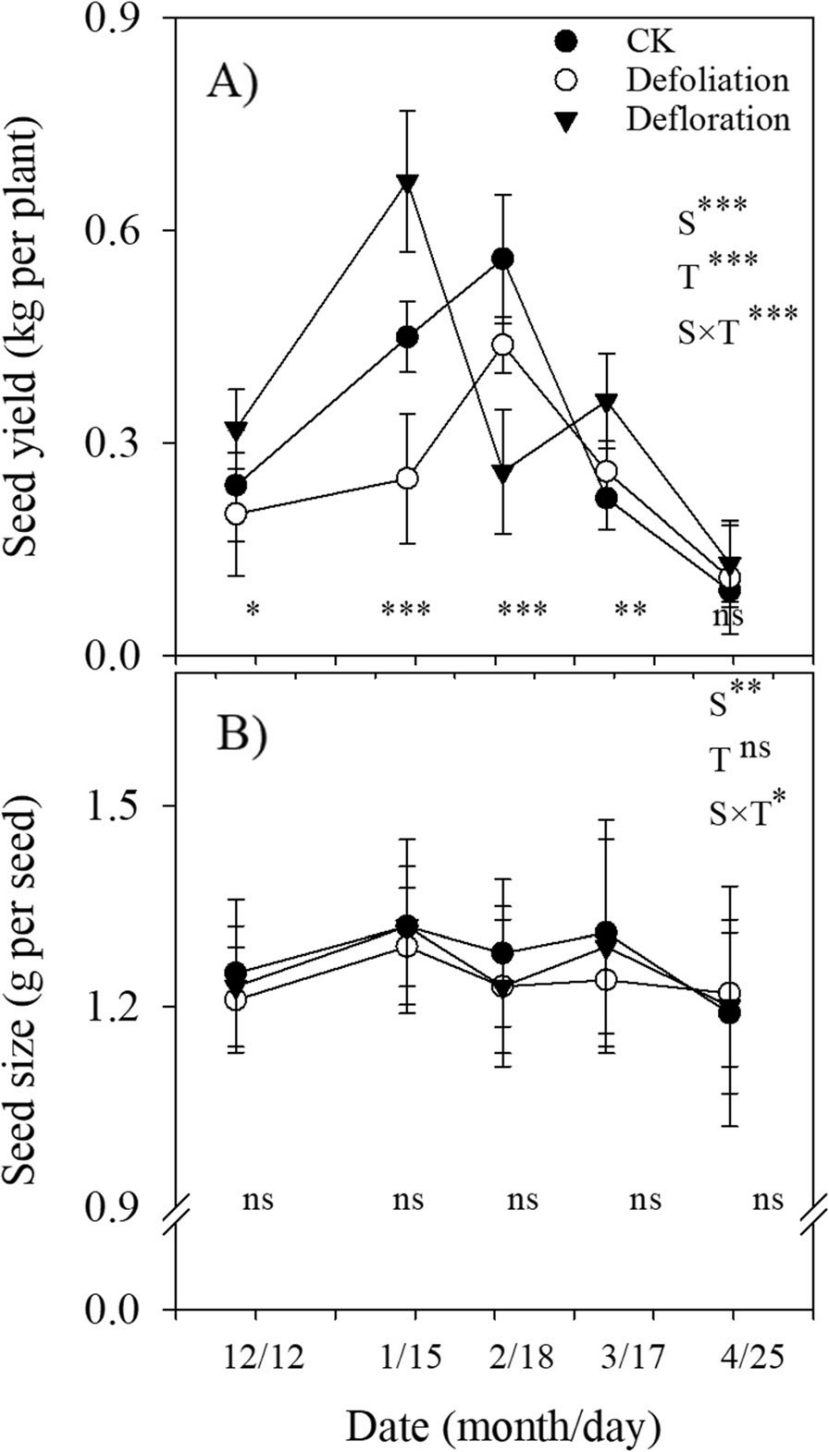
Seasonal dynamics of seed size and seed yield over a growing season of *P. volubilis* plants in response to defoliation and defloration. Significance levels of ANOVAs testing for the effects of season (S), treatment (T) and their interaction are listed for each variable. CK, control. The asterisk denotes significant difference between different treatments for a given harvested date. ns, no significance. * *P* < 0.05; **P < 0.01; ***P < 0.001

**Fig. 5:**
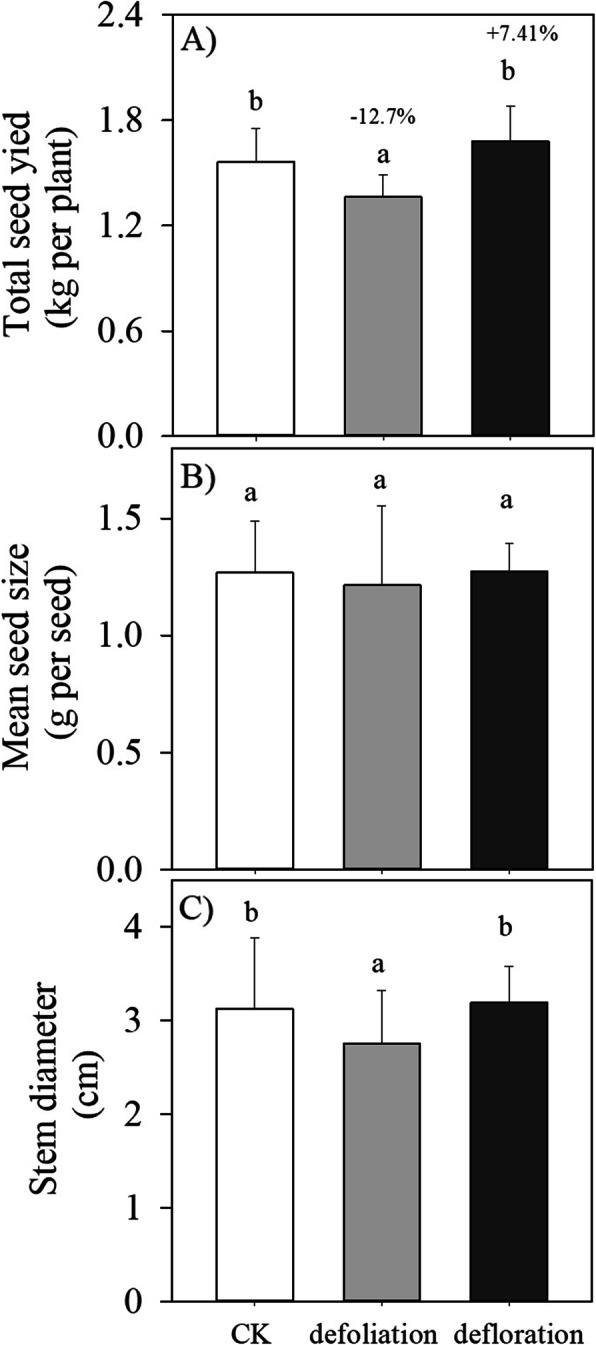
The mean seed size and total seed yield over a growing season, and growth (stem diameter) of *P. volubilis* plants in response to defoliation and defloration. The values with different letters denote significant difference between different treatments at *P* < 0.05 level. CK, control

As a recurrent woody plant that blooms and fruits continuously, fruit (seed) maturation of *P. volubilis* plants showed a marked yearly rhythm, with peak values occurring in the dry season (from December to April) (Fig. 4a). Significant season × treatment interactions affected seed yield, suggesting that seed yield responded to defoliation or defloration differed between different harvested dates. Neither defoliation nor defloration affected the first peak harvest time (i.e., December), but the yield of the peak matured fruits harvested in other dates differed significantly between the different treatments (Fig. 4a). For instance, at time of the second harvest, seed yield in the deflorated plants was much higher than that in the defoliated plants. Throughout the growing season, the highest amounts of mature fruits were obtained at the second and the third harvested date, respectively, in the deflorated and the defoliated plants. But seed size within each harvested date did not differ significantly between three treatments (Fig. 4b). Defloration slightly increased (7.4%), whereas defoliation significantly decreased (− 12.7%) the total seed yield (Fig. 5a), whereas the mean seed size throughout the growing season did not differ between three treatments (Fig. 5b). In addition, defoliation, but not defloration, significantly reduced stem diameter (plant growth) measured at the end of the experiment (Fig. 5c).

**Fig. 6:**
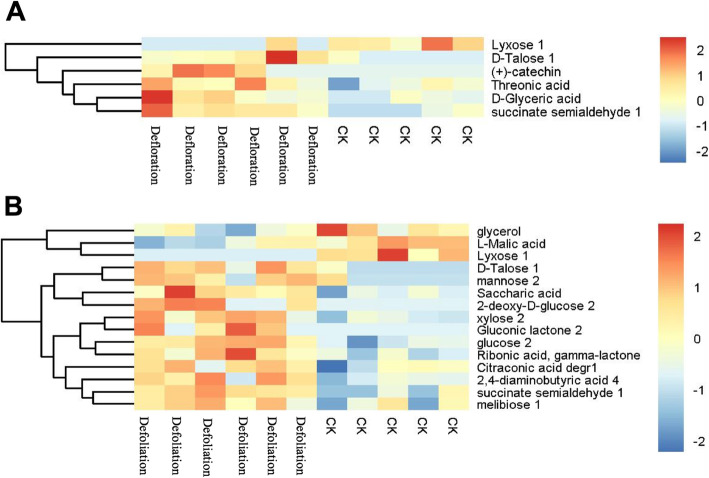
Heatmap of hierarchical clustering analysis for group (**a**) defloration vs. control and (**b**) defoliation vs. control

**Fig. 7:**
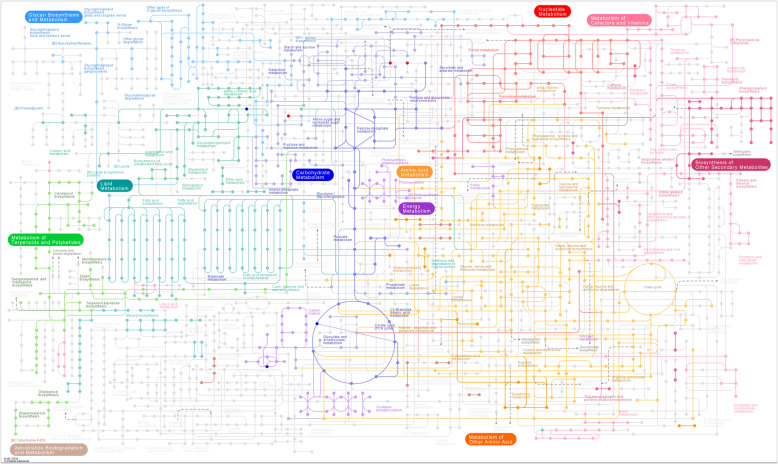
Pathway analysis plot for group defoliation vs. control

**Fig. 8:**
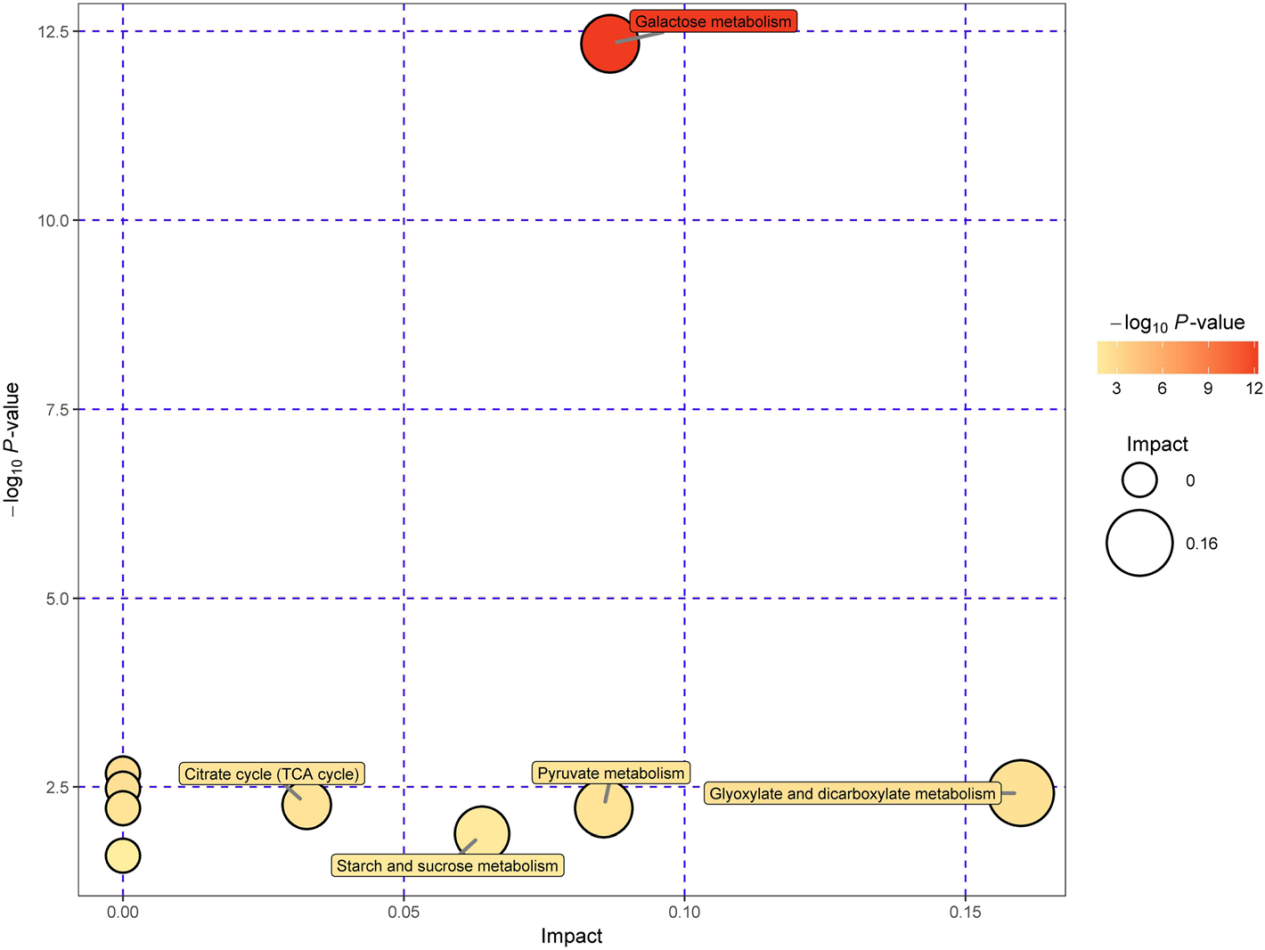
Metabolic pathways with red/blue dots representing the differentially expressed compounds. Warm colors represent upregulation and cold colors represent downregulation

To obtain a better understanding of the potential physiological responses of fruit abortion and development of *P. volubilis* plants in response to source–sink manipulations, we analyzed the metabolites in fruitlets after 24 days of defoliation and defloration applied. Both principal component analysis (PCA) and orthogonal projections to latent structures-discriminant (OPLS-DA) analyses showed that all samples were fall in the 95% Hotelling’s T-squared ellipse, and a significant differentiation occurred in the samples between defoliated and deflorated groups (Fig. S2). That indicated that the source/sink regulation had a substantial effect on the metabolites in fruitlets. Compared with control, defloration increased the contents of five metabolites (D-glyceric acid, threonic acid, D-talose, succinate semialdehyde, (+)-catechin), but decreased only one (i.e., lyxose) (Table 1). Whereas, defoliation increased the contents of thirteen metabolites (D-talose, mannose, xylose, saccharic acid, succinate semialdehyde, ribonic acid, gamma-lactone, 2-deoxy-D-glucose, glucose, melibiose, 2,4-diaminobutyric acid, citraconic acid, gluconic lactone), but decreased the contents of three metabolites (glycerol, L-malic acid and lyxose) (Table 2). Hierarchical clustering showed the associations between samples according to the metabolite profile (Fig. 6a, b). The samples that obtained from each treatment were clustered together. In the defloration treatment, only one metabolite (i.e., lyxose) was separated from the others (Fig. 6a). In the defoliation treatment, three metabolites (i.e., glycerol, L-malic acid, and lyxose) were grouped in a different cluster (Fig. 6b). Analysis of Kyoto Encyclopedia of Genes and Genomes (KEGG) indicated that defloration affected the biosynthesis of secondary metabolites, including glycerate and catechin (Table 3). Whereas, defoliation affected the biosynthesis of secondary metabolites (glucose, malate, and D-glucono-1,5-lactone), galactose metabolism and ABC transporters (glucose, glycerol, mannose, and melibiose), carbon metabolism (malate and D-glucono-1,5-lactone), amino sugar and nucleotide sugar metabolism (glucose and mannose), and pentose phosphate pathway (glucose and D-glucono-1,5-lactone) (Table 4). Furthermore, KEGG pathway analysis indicated that defoliation affected the galactose metabolism, starch and sucrose metabolism, citrate cycle (TCA cycle), pyruvate metabolism, glyoxylate and dicarboxylate metabolism. But we did not find the significant enriched KEGG pathway in the defloration group (Table 5, Fig. 7). Supporting this assumption, the KEGG pathway analysis indicated that defoliation downregulated the TCA cycle, lipid metabolism, and carbohydrate metabolism in fruitlets (Fig. 8).

**Table 1:** Differential metabolite contents in fruitlets between the control and the deflorated group

Peak	Similarity	R.T.	Count	Mass	MEAN H	MEAN CK	VIP	*P*-VALUE	Q-VALUE	FOLD CHANGE	LOG_FOLD CHANGE
D-Glyceric acid	824	12.6638,0	17	189	0.401823	0.223858	2.149468	0.049167	0.727878	1.794990	0.8439755
D-Talose 1	795	18.7309,0	13	400	0.010151	0.001141	2.565858	0.021771	0.727878	8.900472	3.1538819
Threonic acid	774	15.2487,0	17	292	0.155878	0.109859	1.593730	0.048319	0.727878	1.418901	0.5047740
succinate semialdehyde 1	646	10.9787,0	14	89	0.019193	0.003556	2.039284	0.002755	0.727878	5.396574	2.432044
Lyxose 1	631	16.3242,0	6	307	0.000715	0.004001	2.662419	0.013554	0.727878	0.178766	−2.4838523
(+)-catechin	356	27.6272,0	7	369	0.001036	3.4742E-09	2.279293	0.042309	0.727878	298,172.8	18.185789

**Table 2:** Differential metabolite contents in fruitlets between the control and the defoliated group

Peak	Similarity	R.T.	Count	Mass	MEAN Y	MEAN CK	VIP	P-VALUE	Q-VALUE	FOLD CHANGE	LOG_FOLD CHANGE
glycerol	834	11.9581,0	17	205	0.216576	0.301440	1.657182	0.042461	0.430477	0.718473	−0.476994
glucose 2	808	18.9806,0	17	205	2.37325	1.651711	2.317185	0.000408	0.085609	1.436844	0.522903
D-Talose 1	795	18.7309,0	13	400	0.010801	0.001141	2.174023	0.001439	0.120522	9.470816	3.243489
mannose 2	793	18.8215,0	9	69	0.066114	0.014789	1.649186	0.033848	0.411916	4.470335	2.160383
L-Malic acid	770	14.5738,0	15	233	0.750475	1.840440	1.194873	0.011054	0.296846	0.407770	−1.294174
xylose 2	731	16.2726,0	14	307	0.011179	0.005439	2.104359	0.005713	0.243256	2.05542	1.039436
Saccharic acid	713	19.7989,0	16	333	0.032370	0.012739	1.11860	0.006400	0.252564	2.541020	1.34541
succinate semialdehyde 1	646	10.9787,0	14	89	0.014395	0.003556	1.846162	0.001179	0.112956	4.047604	2.017068
Lyxose 1	631	16.3242,0	6	307	3.2168E-09	0.004001	2.621495	0.007627	0.266172	8.0399E-07	−20.246319
Ribonic acid, gamma-lactone	545	16.7769,0	17	220	0.014783	0.007332	2.119316	0.006766	0.256972	2.016307	1.011715
2-deoxy-D-glucose 2	539	17.762,0	5	276	0.04742	3.4742E-09	1.912997	0.030747	0.403271	13,650,449.3	23.70245
melibiose 1	466	27.6702,0	15	204	0.003359	0.001637	1.357949	0.049263	0.441266	2.05163	1.036769
2,4-diaminobutyric acid 4	426	16.0886,0	17	174	0.028417	0.012646	1.807167	0.010068	0.289573	2.247154	1.168099
Citraconic acid degr1	330	11.7363,0	17	89	0.004631	0.003053	1.422573	0.040643	0.427094	1.516855	0.601083
Gluconic lactone 2	204	18.8577,0	7	201	0.024234	3.4742E-09	2.007634	0.037813	0.421305	6,975,510.2	22.73387

**Table 3:** KEGG analysis in fruitlets in the deflorated group

KEGG Pathway	Compound
ath01110 Biosynthesis of secondary metabolites - *Arabidopsis thaliana* (thale cress) (2)	cpd:C00258 D-Glycerate; cpd:C06562 (+)-Catechin

**Table 4:** KEGG analysis in fruitlets in the defoliated group

KEGG Pathway	Compound
ath01100 Metabolic pathways - Arabidopsis thaliana (thale cress) (5)	cpd:C00031 D-Glucose; cpd:C00116 Glycerol; cpd:C00149 (S)-Malate; cpd:C00159 D-Mannose; cpd:C00198 D-Glucono-1,5-lactone
ath00052 Galactose metabolism - Arabidopsis thaliana (thale cress) (4)	cpd:C00031 D-Glucose; cpd:C00116 Glycerol; cpd:C00159 D-Mannose; cpd:C05402 Melibiose
ath02010 ABC transporters - Arabidopsis thaliana (thale cress) (4)	cpd:C00031 D-Glucose; cpd:C00116 Glycerol; cpd:C00159 D-Mannose; cpd:C05402 Melibiose
ath01110 Biosynthesis of secondary metabolites - Arabidopsis thaliana (thale cress) (3)	cpd:C00031 D-Glucose; cpd:C00149 (S)-Malate; cpd:C00198 D-Glucono-1,5-lactone
ath01200 Carbon metabolism - Arabidopsis thaliana (thale cress) (2)	cpd:C00149 (S)-Malate; cpd:C00198 D-Glucono-1,5-lactone
ath00520 Amino sugar and nucleotide sugar metabolism - Arabidopsis thaliana (thale cress) (2)	cpd:C00031 D-Glucose; cpd:C00159 D-Mannose
ath00030 Pentose phosphate pathway - Arabidopsis thaliana (thale cress) (2)	cpd:C00031 D-Glucose; cpd:C00198 D-Glucono-1,5-lactone

**Table 5:** KEGG pathway enrichment analysis in fruitlets in the defoliated group

Pathway	Total	Hits	Raw p	-log(p)	Holm adjust	FDR	Impact
Galactose metabolism	26	4	4.3984E-06	12.334	0.00038266	0.00038266	0.08673
Glycerolipid metabolism	13	1	0.068704	2.678	1	1	0
Fructose and mannose metabolism	16	1	0.083971	2.4773	1	1	0
Glyoxylate and dicarboxylate metabolism	17	1	0.089012	2.419	1	1	0.15986
Citrate cycle (TCA cycle)	20	1	0.10399	2.2634	1	1	0.03272
Carbon fixation in photosynthetic organisms	21	1	0.10894	2.217	1	1	0
Pyruvate metabolism	21	1	0.10894	2.217	1	1	0.08561
Starch and sucrose metabolism	30	1	0.15241	1.8812	1	1	0.06394
Amino sugar and nucleotide sugar metabolism	41	1	0.20307	1.5942	1	1	0

## Discussion

### Source–sink manipulations do not regulate leaf photosynthesis and morphology

We find no evidence that the regulation of leaf photosynthesis compensated for leaf or fruit loss in the defoliated or deflorated plants (Fig. 1a). A lack of photosynthetic upregulation and reduced leaf area in the defoliated plants were also reported in black oak trees (*Quercus velutina* Lam.) [28]. The physiological mechanism that drives the change in *P*_n_ following defoliation appears to vary widely between species [14, 29, 30]. Although leaf N content increased by defoliation, the stable *P*_n_ was probably due to the changes in within-cell partitioning of N within the leaf to decrease Rubisco levels (thus lower PNUE; Fig. 1e) [31]. Moreover, low soil nutrient availability may restrict the potential for compensatory photosynthesis and growth after defoliation [13]. But this does not seem to be the case in our study, as all plants were sufficiently supplied with fertilizer to avoid growth limitation by soil nutrients. Alternatively, having higher leaf WUEi values (Fig. 1d, but [6]), the defoliated plants were less water-stress than those of control plants, largely owing to a reduced transition surface. It was also reported that, in a young *Eucalyptus nitens* plantation, the aboveground biomass production per unit transpiration (i.e., stand water-use efficiency) was increased by shoot pruning [32]. No significant variations in photosynthetic rate in the deflorated plants were found compared with control, suggesting that the sink effect was moderate in 2.5-year-old *P. volubilis* plants bearing approximately hundred fruits.

Across all seasons, the lowest SLA value found in the dry season supported the consumption that SLA in leaves that develop under water-deficit conditions was generally lower than those develop under wet conditions [31]. Whereas SLA did not change greatly between different source or sink manipulations (Fig. 1f). It was found that severe fruit pruning did not change leaf morphology (leaf size and area) in oil palm (*Elaeis guineensis*) [4]. Therefore, a relatively stable SLA of *P. volubilis* plant in response to disturbance is important to maintain leaf structural function and adapt to drought [25, 27].

### C and N differently respond to source–sink manipulations

Carbohydrates stored in woody tissues of perennial plants are essential for a sustainable crop production because of their role in nutrient (mainly N) uptake and assimilation, leaf area formation, and inflorescence induction, particularly under stress situations [2]. Since *P. volubilis* plants have extremely small root biomass fraction (ca. 6%) and store only very little NSC in roots [27, 33], vegetative aboveground tissues have to assume these functions. The highest and lowest NSC contents occurred in the middle-wet season and in the dry season, respectively (Fig. 2d), reflecting the phenomenon of ‘refill’ and ‘carbon depletion’ during the wet and dry season, respectively. Water stress in the dry season can cause substantial inhibition of photosynthesis and have necessitated a decrease in carbohydrate mobilization from storage tissues [27, 34]. Whereas in a pot experiment with four grapevine varieties, the increased rather than decreased carbohydrates in wood tissues was found under prolonged drought because of the decreased demand of aboveground growth [35].

NSC contents (especially sugar) were sharply reduced within a short period (three weeks) after defoliation applied in the early-wet season, but were completely recovered in the late-wet season and were maintained in the dry season (Fig. 2b, d). Our findings are consistent with the consumption that fast-growing species (e.g., *P. volubilis*), typically having low leaf construction costs and more flexible growth strategies, respond rapidly to defoliation, through changes in carbohydrate contents [36]. Defoliated plants reduced NSC contents, although temporally, either by reducing the photosynthetic apparatus and/or by triggering new foliage growth, but not by the compensatory photosynthesis observed (Fig. 1a). On the other hand, defloration increased available resources through sink limitation, as indicated by the continuous increase in NSC reserves (Fig. 2d). This was probably the cause of the less resource competitions between the remaining fruits and also between vegetative and reproductive tissues. Thus, carbohydrate reserves in the vegetative tissues of *P. volubilis* plants served to buffer sink–source imbalances that may result from temporary reductions in demand for assimilates (e.g., defloration) or shortfalls in carbon assimilation (e.g., defoliation) [9, 10, 37].

After defoliation, C and N limitations are assumed to result in the decreased plant growth and even plant mortality, especially in evergreen species that they have abundant nutrition reserves (e.g., N and P) in leaves year-round [1, 30]. Compared with control, the defoliated *P. volubilis* plants had a temporally reduced NSC contents and lower long-term growth (Fig. 5c). This was consistent with the result of a large-scale manipulative field experiment in a hybrid poplar plantation, where repeated defoliation lead to long-term growth decline but only transient C storage reduction occurred [37]. On the other hand, defoliation lead to the increased N contents in both remaining leaves (Fig. 1c) and branches (Fig. 3a). The incensement of the internal N availability contributed to the initiation of new axes and the functional biomass partition between woody tissues and/or the enhanced N uptake from soils increased N contents in plant tissues [2, 7, 37, 38]. Thus, C limitation, but not N remobilization, is a source-driven growth process in *P. volubilis* plants [cf. 1]. But for a winter-deciduous temperate adult tree (*Nothofagus pumilio*), the greatly reduced plant growth induced by extreme defoliation was due to growth limitation (i.e., preventative C allocation to storage), rather than insufficient C or N availability [15]. A more integrated understanding of the possible shifts in C and/or N limitation on growth and the yield that occur during the lifetime of *P. volubilis* plants, is required.

### Source–sink manipulations differently regulate the reproductive traits

After defoliation, either the advanced or delayed budburst was found in some temperate deciduous fruit trees (e.g., peach, [39]; walnut, [40]), because of the dose-dependent response of the supply of sugars (especially sucrose) involved in budburst processes. For the recurrent woody plants that bloom and fruit continuously in tropical areas, flowering and fruit maturation date show a marked yearly rhythm but the control of their periodicity is not well understood. Largely determined by temperature and photoperiod in plants without a vernalization requirement within a growing season [31], the phonological development of *P. volubilis* plants, i.e., initial peak mature time, was not affected by source–sink regulations (Fig. 4a), contrasted to the result in the long-term exposure to shade [41]. But the dates of the most peak mature fruits harvested differed between different treatments. The highest mature fruits were harvested earlier in the deflorated plants than the control; the reverse was true in the defoliated plants (Fig. 4a). A decrease in assimilate supply, due to leaf removal, might increase early fruit abortion thus delayed fruit maturation in evergreen trees [42].

Combined with a small fraction of fruit sets (< 40%, Fig. 3 [33];), the well-developed reproductive tissues (i.e., relatively large flower numbers per plant [25];) indicated that flower initiation did not limit the number of fruits produced. Fruit retention and development are thus the limiting factors for the yield of the wind-pollinated *P. volubilis* plants. Fruit (seed) number is a dominant factor for seed yield because seed size in each harvesting date and throughout the growing season did not differ significantly between three treatments (Fig. 4b; Fig. 5b). Along with our previously studies that fruit (seed) number, rather than seed size, responded greatly to agricultural management practices (e.g., fertilizers, irrigation and plant density [25–27];), our results supported that, from an evolutionary perspective, plants would have much less plasticity in seed weight than in seed number [43]. Increased fruit (seed) production following artificial defoliation or herbivory (i.e., overcompensation) has been found in some wild plant species and herb crops (e.g., potatoes), but the potential negative or zero effect of source-reducing on crop yield depends on the studied crop species and plant size, environmental conditions, and the frequency and intensity of defoliation [44]. Fruit development can be supplied with C imported either from current photoassimilates of neighboring branches or from C reserves stored in the woody tissues. Defloration slightly increased, whereas defoliation greatly decreased the total seed yield (Fig. 5b). This implied that source–sink regulations affected fruit (seed) yield mainly by decreasing flower bud number and/or inducing fruit abortion, rather than individual fruit growth (size).

The alteration of the whole-plant carbon balance induced by source–sink regulations affect nutrition and carbon reserve in leaves and the reproductive tissues (flowers and fruitlets), leading to abscission [10, 42]. Fruit abortion not only depends on the source strength but also on the sink strength of competing tissues [9, 11, 45]. Within a plant system, the smallest and less developed fruitlets generally undergo abortion, as the strongest fruitlets are positively selected against the weakest ones [5]. In our study, defoliation reduced, but defloration increased the percentage of fruit abortion, especially in the earlier reproductive stage (Fig. 3). Thus, high fruit set of *P. volubilis* plants was involved an increased photosynthetic input and thus increased resource limitation (no defoliation), and less sink strength of competing reproductive tissues (deflorated plants). It was also reported that carbohydrate shortage, especially sugar, leads to dramatically accelerated fruitlet abortion in lychee, a recurrent tropical fruit tree [46]. Generally, plants with a larger plant size (stem diameter) had the higher total seed yield across all treatments (Fig. 5c), indicating that the amount of stored resources (i.e., carbohydrate and N) from maternal plants mainly determine the seeds (fruits) numbers during the reproductive stage [27, 41]. Compared with control, the reduced amount of current and stored carbohydrates by defoliation during the reproductive stage may restrict the numbers of fruits (fruit load; but see olive tree [12]), especially in the dry season when water was limited [25]. Carbohydrates (especially sugar) play a key role in flower bud formation and their levels can be directly correlated with floral induction in fruit trees [11]. Our results indicated that fruit retention and thereafter mature fruit number, rather than individual seed development (size), is strongly source-limited in *P. volubilis* plants [c.f. 27]. However, owing to a complex carbohydrate and hormone signalling crosstalk controlling flower/fruitlet abscission [2], we still cannot know whether the varied mature fruit number (load) in response to source–sink manipulations is due to a direct effect of fruit set, or an indirect effect caused by changes on the total fruit number per plant.

The source or sink regulation had a substantial effect on the metabolites in fruitlets, as a significant differentiation occurred in the samples between defoliated and deflorated groups (Fig. S2). Compared with control, fewer metabolites in fruits showed relatively lower fluctuations in the defoliated plants than in the deflorated plants, especially for the secondary metabolites (Tables 1, and 2). Given that defloration is known to reduce the competition for carbohydrate source and thus promote fruit size and carbohydrate content in several fruit trees including peach [47] and apple [48], it can be expected that the assimilated photosynthetic carbohydrate in the deflorated *P. volubilis* plants might be diverged to growth rather than to secondary metabolite synthesis in fruitlets. Defoliation downregulated the TCA cycle and carbohydrate metabolism in fruitlets (Fig. 8), resulting in carbon starvation and insufficient energy metabolism, thus inhibiting the growth and enhancing fruit abortion [11, 46]. Moreover, at the whole-plant level, regulation of primary metabolism determines the C:N balance and also affects sink strength [3, 21]. The decreased content of malic acid by defoliation may limit the energetic cost for fruit development, as it serves as a crucial intermediate involved in several metabolic pathways, such as TCA cycle, amino acid metabolism and biosynthesis of plant secondary metabolites [11, 20]. Interestingly, compared with control, both defoliation and defloration decreased lyxose content (Tables 1, and 2). Direct regulatory role of this compound is unclear. It was found that xylose is a major component of the pericarp cell walls of tomato fruits [22]; the metabolism of this hemi-cellulose is key for wall loosening linked with cell expansion [49]. Moreover, fruit xylose was connected to leaf trehalose, possibly linking leaf sugar sensing to fruit expansion [22]. On the other hand, the lipid production in oilseed crops requires great demand of carbon sources, such as sugars produced by photosynthesis in leaves [50]. Defoliation, but not defloration, reduced the content of glycerol (Table 2), a product from lipid metabolism, implying that the reduced lipid accumulation occurred in seeds. Overall, this study indicated potentially important metabolites that are correlated to fruit abortion and fruit development would provide a basis for further study on the process of fruit maturation during various developmental stages.

## Conclusions

To our knowledge, this is the first study to infer the effect of source–sink regulations on the physiological and reproductive traits of a recurrent evergreen woody crop, *P. volubilis*. Compared with control, although the temporally reduced NSC contents (especially sugar) can be fully recovered, the decreased growth in the defoliated plants was caused by C limitations, rather than insufficient N availability. Defloration increased, whereas defoliation decreased the total seed yield throughout the growing season, which was determined by the mature fruit number, rather than fruit size. Source or sink regulation had a substantial influence on the metabolites in fruitlets. Slightly defloration, but not defoliation, is recommended for both the yield and carbohydrate and lipid accumulation in fruits in the strong source-limited *P. volubilis* plants. A long-term research work is needed to understand how whole plant physiology are regulated by source–sink relationships under environmental and developmental constraints between seasonal and yearly variations for this recurrent crop, which has a complex phenology and is substantially affected by defoliation [2, 3].

## Methods

### Experimental site and plant material

The experiment was conducted in Xishuangbanna Tropical Botanical Garden (21^0^56′N, 101^0^15′E, 560 m asl), Chinese Academy of Sciences. Owing to the southwest monsoon in Xishuangbanna, distinct wet (from May to October) and dry (from November to April) season occurred. The average annual rainfall there is 1500 mm, ca. 85% of which falls in the wet season. The average annual temperature is 22.9 °C. The physico-chemical traits in soils of the top layer (0–20 cm) were described in our previous work [27]. Meteorological conditions in the study area during the experimental period are summarized in Fig. S1, which was available as Supplementary Data Online.

Seeds of *P. volubilis* plants were obtained from a commercial company in south America (Namaskar S.A.C. Inc., Lima, Peru). *P. volubilis* plants flower about six months after seeds were sown. The field experiments were conducted in *P. volubilis* plantations using 2.5-year-old plants, which were cultivated at intra- and inter-row spacing of 2.0 m and 2.0 m, respectively. A split-plot design with randomized complete blocks was arranged; three replications in a 2 m × 44 m sized plot were used in each treatment*.* Compound fertilizer (150 kg per ha) was spread in early June each year, according to our previous agriculture management practice [25]. Due to the non-self-supported adult liana, all *P. volubilis* plants were supported by steel wire to a height of 1.6 m. To avoid edge effects, only the central healthy plants in the plots were selected.

### Source–sink manipulations

A total of 8–10 plants were selected within each plot. The source–sink manipulation took place between June 28 and 2 July 2016 (i.e., in the early-wet season when plants grow fast) and was performed randomly. Prior to defoliating and defoliating, for each plant, the numbers of the canopy mature leaves (net C sources) and fruitlets (diameter < 1 cm, net C sink tissues) was counted. In each block, plants were randomly assigned to one of the treatments: defoliation (randomly cutting ca. 10% canopy mature leaves in each branch per plant), defloration (randomly thinning 10% fruitlets in each branch per plant), and control (neither defoliation nor defloration). Buds in the defoliated or deflorated plants were unaffected. A total of ten to twelve plants were monitored within each treatment in three selected plots.

### Photosynthetic traits and chemical measurements

Gas exchanges were made in the morning between 9:00 and 11:30 with a Li-Cor LI-6400 system, in August 20 (middle wet season), October 14 (late wet season) in 2016, and April 12 (dry season) in 2017, respectively. Four to six plants were selected per treatment for the photosynthetic measurements in each season; one fully-expanded, mature canopy leaf was measured per plant. The net light-saturated photosynthetic rate (*P*_*n*_) and stomatal conductance (*g*_s_) were measured under the light-saturating irradiance and ambient CO_2_ concentration as described previously [40]. Subsample of leaves was scanned by a desktop scanner, and the image was analysed for leaf area. After photosynthetic measurements, leaves and 1-year-old branches in each season were harvested and were then weight after drying at 70 °C for 48 h, and was used for the measurement of N content. Specific leaf area (SLA = leaf area/leaf mass), instantaneous water-use efficiency (WUEi = *P*_*n*_/gs) and photosynthetic N-use efficiency (PNUE = *P*_*n*_/N) were then calculated.

Total N content of leaf or branch sample was determined using the standard micro-Kjeldahl method. The dried branch tissue was also analysed for NSC, defined as the sum of starch and total soluble sugar, following the enzymatic digest and ultraviolet spectrophotometry methods modified from DuBois et al. (1956) [51]. We focused mainly on carbohydrate content, since a shift in carbohydrate content relative to controls following disturbance is indicative of C storage being used for regrowth [25, 26].

### Growth and reproductive traits

Stem diameter of each individual plant at 10 cm above soil level was measured with a calliper to assess plant growth at the end of April 2017. A total of 30 female flowers with their stigmata open were randomly tagged in each plot in late Nov. 2016 and Jan. 2017, respectively; formation of fruits was then counted within 3 weeks. The percentage of fruit set was calculated as the number of set fruits divided by the number of female flowers× 100.

Mature fruits were harvested manually and repeatedly five times in each individual plant throughout the fruit maturing period; fruit dry mass per plant was measured at each harvest. At each harvest, subsamples of mature fruits were peeled and seed dry mass (size) was weighted. Seed yield in each harvested date and the total seed yield (per plant) throughout a growing season were then calculated as we descripted previously [24, 26].

### Metabolite profiling analysis in fruitlets

For the metabolite profiling analysis in fruitlets after 24 days of defoliation or defloration applied, samples of the canopy fruitlets (diameter = 1–1.2 cm) were collected in 9:30–10:30 in the sunny morning and immediately frozen in liquid nitrogen and stored at − 80 °C; six biological replications were used in each treatment. Using an Agilent 7890 gas chromatograph system (Agilent, California, Palo Alto, USA) coupled with a Pegasus HT time-of-flight mass spectrometer, metabolite extractions, derivatization and gas chromatography time-of-flight mass spectrometry (GC-TOF MS) analyses from the fruitlets (100 mg FW) were carried out as previously described by Lisec et al. (2006) [52] with some modifications. The details of the measurements and data analysis were described in Supplementary Text Online.

### Statistical analysis

Two-way ANOVAs with season (S) and treatment (T) as the main factors were used for the morphological, physiological and reproductive variables. Then a Tukey HSD post-hoc test was applied within each factor. To satisfy the assumptions of ANOVAs, the normality and homogeneity of data were checked; when necessary, a log_10_- or square-root transformation was used. All data were analyzed by SPSS software (version 23.0).

## Supplementary Information

**Additional file 1.** Supplementary material related to this article can be found in the online version.

## Data Availability

The datasets generated during the current study are available from the first author on reasonable request.

## Abbreviations

*P*_n_: Net light-saturated photosynthetic rate
g_*s*_: Stomatal conductance
WUEi: Instantaneous water-use efficiency
PNUE: Photosynthetic N-use efficiency
NSC: Nonstructural carbohydrates
TCA: Tricarboxylic acid
OPLS-DA: Orthogonal projections to latent structures-discriminant
PCA: Principal component analysis
KEGG: Kyoto Encyclopedia of Genes and Genomes
