# Supplementary material for: Source–sink manipulations differentially affect carbon and nitrogen dynamics, fruit metabolites and yield of Sacha Inchi plants
Source: BMC Plant Biol. 2021 Mar 30;21:160. doi: 10.1186/s12870-021-02931-9 (PMC8011213; doi:10.1186/s12870-021-02931-9)
Supplement: Supplementary file 1 — Additional file 1. Supplementary material related to this article can be found in the online version. [file 12870_2021_2931_MOESM1_ESM.pdf]

Supplemental files:

1. Supplementary text: Material and Method details

Metabolite measurements and GC-TOF-MS analyses

Gas chromatography–time of flight-mass spectrometry (GC-TOF-MS) analysis was performed using an Agilent 7890 gas chromatograph system coupled with a Pegasus HT time-of-flight mass spectrometer. The experimental procedure for extract preparation was performed as described previously (Lisec et al., 2006). The system utilized a DB-5MS capillary column coated with 5% diphenyl cross-linked with 95% dimethylpolysiloxane (30 m×250 μm inner diameter, 0.25 μm film thickness; J&W Scientific, Folsom, CA, USA). A 1μL aliquot of the analyte was injected in splitless mode. Helium was used as the carrier gas, the front inlet purge flow was 3 mL min^{-1}, and the gas flow rate through the column was 1mL min^{-1}. The initial temperature was kept at 80°C for 1min, then raised to 290°C at a rate of 10°C min^{-1}, then kept for 12 min at 290°C. The injection, transfer line, and ion source temperatures were 280, 295, and 220°C, respectively. The energy was -70eV in electron impact mode. The mass spectrometry data were acquired in full-scan mode with the m/z range of 50-600 at a rate of 12 spectra per second after a solvent delay of 8.45 min. Metabolites for GC-TOF-MS were extracted and analysed using a method described previously (Osorio et al., 2012) with modifications. Chroma TOF 4.3X software of LECO Corporation and LECO-Fiehn Rtx5 database were used for raw peaks exacting, the data baselines filtering and calibration of the baseline, peak alignment, deconvolution analysis, peak identification and integration of the peak area (Tobias et al., 2009).

The metabolic data were standardized using Simca-P software (version 11.0, http://www.umetrics.com/simca). Principal component analysis (PCA) was performed to display variation patterns in all the metabolite data. An orthogonal projection to latent structured discriminant analysis (OPLS-DA) was used to make inferences about those metabolites with highest influential weighting in response to defoliation or defloration treatment (Wiklund et al., 2008). Quantitative normalization within replicates was achieved, and the software of MetaboAnalyst 4.0 (http://www.metaboanalyst.ca/faces/ModuleView.xhtml) was used to build heat map diagrams and metabolite category (Chong et al., 2018). The final model included the watering class summarizing the correlated variation in the predictive component (t_p), and two more orthogonal components (1+2) containing the uncorrelated variation that was not related to the separation of the different treatments (to). Response variables were log transformed to meet the assumptions of normality and homogeneity of variance for the univariate analysis, and were pareto-scaled before multivariate analysis. We used S plots to discriminate those metabolites and functional traits with highest loading in the response to different treatments. A heat-map from the z-transformed median values for each metabolite in fruitlets from both the control and defoliation or defloration treatment was constructed to estimate similarity of the metabolic response among the different families. Only metabolites that showed a significant variation

according to family in the univariate analysis were included in the heat-maps. Q mBROLE was used to determine which metabolic pathways were being affected by defoliation or defloration. By calculating the number of metabolites affected that occur in a particular pathway mBROLE can determine which metabolic pathways were most affected under a condition (Chagoyen and Pazos, 2011). From the OPLS-DA models comparing control and defoliation or defloration, metabolites with a VIP > 0.8 were submitted using their KEGG IDs (Ogata et al., 1999). Pathways that were enriched with a false discovery rate adjusted p-value <0.05 were accepted as affected by defoliation or defloration. Metabolic pathways that are commonly found by this type of analysis due to overbroad interpretation of KEGG pathways were removed. The databases including KEGG (http://www.genome.jp/kegg/) and NIST (http://www.nist.gov/index.html) was utilized to search for the pathways of metabolites. MetaboAnalyst, which use the high-quality KEGG metabolic pathway as the backend knowledgebase, for pathway analysis (http://www.metaboanalyst.ca).

References

Chagoyen M, Pazos F. 2011. MBRole: enrichment analysis of metabolomic data. Bioinformatics 27, 730–731.

Lisec J, Schauer N, Kopka J, Willmitzer L, Fernie AR. 2006. Gas chromatography mass spectrometry-based metabolite profiling in plants. Nat. Protoc. 1, 387–396.

Ogata H, Gota S, Sato K, Fujibuchi W, Bono H, Kanehisa M. 1999. KEGG: Kyoto Encyclopedia of Genes and Genomes. Nucleic Acids Res. 27, 29–34.

Osorio S, Do PT, Fernie AR. 2012. Profiling primary metabolites of tomato fruit with gas chromatography/mass spectrometry. Methods Mol. Biol. 860, 101–109.

Tobias K, Gert W, Yup LD, Yun L, Mine P, Sevini SF, Oliver F. 2009. FiehnLib – mass spectral and retention index libraries for metabolomics based on quadrupole and time-of-flight gas chromatography/mass spectrometry. Anal. Chem. 81, 10038–10048.

Wiklund S, Johansson E, Sjöström L, et al. 2008. Visualization of GC/TOF-MS-based metabolomics data for identification of biochemically interesting compounds using OPLS class models. Anal. Chem. 80, 115–122.

2. Supplementary data:

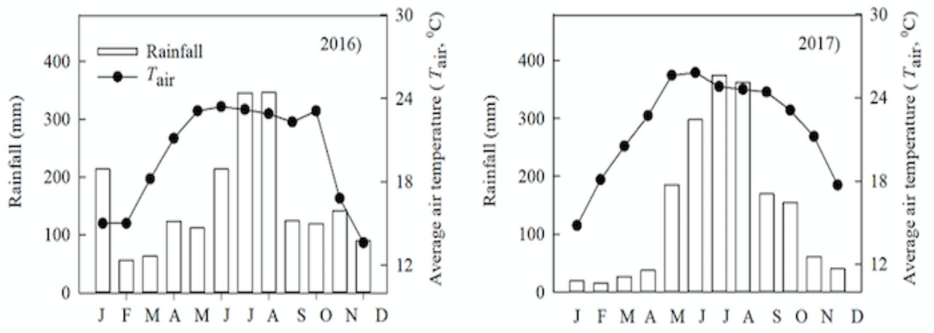

Fig. S1. Seasonal changes in monthly precipitation (bars), mean air temperatures (T_{air}; ●) in the experimental period.

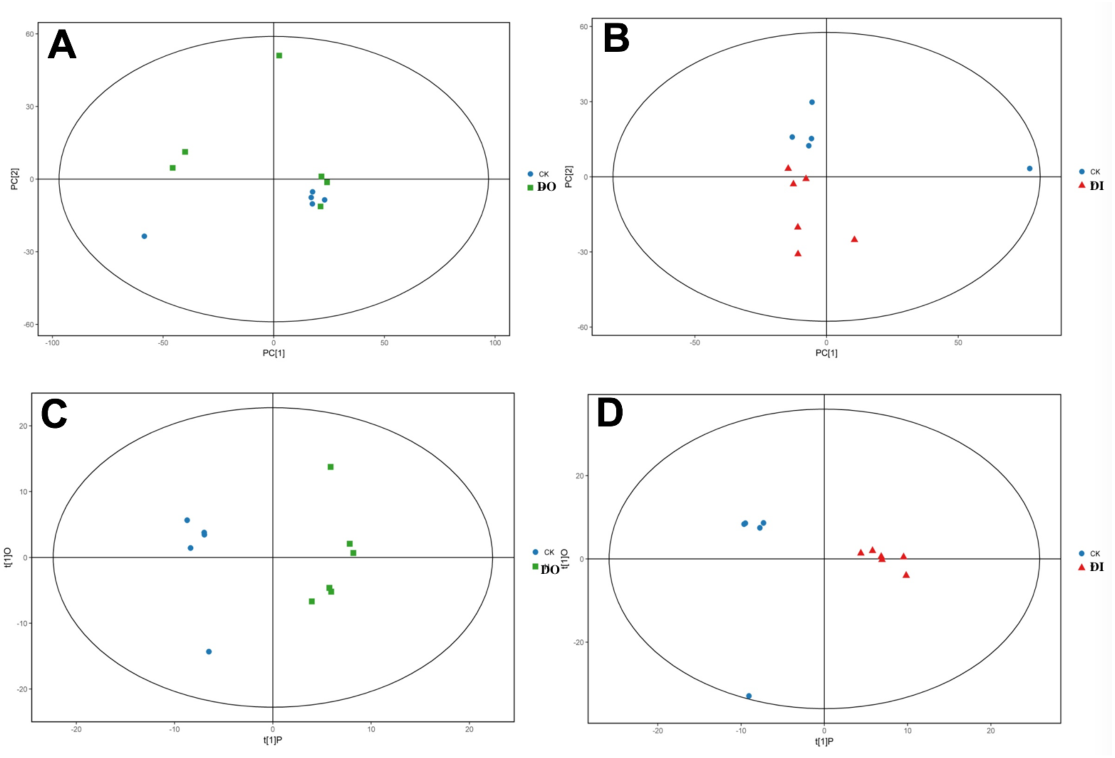

Fig. S2. (A) Score scatter plot of PCA model for group defloration-treatment vs. control. (B) Score scatter plot of OPLS-DA model for group defloration-treatment vs. control. (C) Score scatter plot of PCA model for group defoliation-treatment vs. control. (D) Score scatter plot of OPLS-DA model for group defoliation-treatment vs. control. DO, defloration; DI, defoliation; CK, control.
